# Supplementary material for: Advancing science or advancing careers? Researchers’ opinions on success indicators
Source: PLoS One. 2021 Feb 11;16(2):e0243664. doi: 10.1371/journal.pone.0243664 (PMC7878066; doi:10.1371/journal.pone.0243664)
Supplement: S1 File — (PDF) [file pone.0243664.s001.pdf]

S1 FILE

PRINTOUT OF THE SURVEY

Start of Block: Introduction

Welcome to our Survey for the project Re-SInC.

The survey aims to rethink research assessments and research careers. We want to know what **you** think.

You may find the full information for participation here.

To make this short, we thought we would give you three reasons for taking part in this survey!

- We need your input to know what really matters in research.
- It will only take 15-20 minutes of your time (we tested it with a few people, and it never took more than 16 minutes!).

You will help a very grateful PhD student graduate!

Note on ethics and privacy: This project has been approved by the Medical Ethics Committee of Hasselt University, protocol number CME2019/O35. Answers to the survey will be **fully confidential**, and **no identifiable information** (e.g., IP adresses, emails, etc.) is collected in the survey. The dataset, which contains **no identifiable information**, will be made public when findings are published.

Closing date: October 31st 2019
If possible, use a computer to complete this questionnaire.
If you only have a phone at hand, you should use the landscape mode.

End of Block: Introduction

Start of Block: Demographics

Before we start, we would like to get to know you!

You are a...

- o PhD Student
- o PostDoc / Non-tenure-track position
- o Tenure-track researcher / Professor
- o Tenured researcher / Full professor
- o I was a researcher in the past, but moved to another career
- o Other (specify) ____

Display This Question:
If Before we start, we would like to get to know you! You are a... = PhD Student
Or Before we start, we would like to get to know you! You are a... = PostDoc / Non-tenure-track position
Or Before we start, we would like to get to know you! You are a... = Tenure-track researcher / Professor
Or Before we start, we would like to get to know you! You are a... = Tenured researcher / Full professor
Or Before we start, we would like to get to know you! You are a... = Other (specify)

You have been in this position for ...

▼ less than one year ... 9 years or more

Display This Question:
If Before we start, we would like to get to know you! You are a... = I was a researcher in the past, but moved to another career

How long ago did you stop being a researcher?

▼ less than one year ... 9 years or more

Display This Question:
If Before we start, we would like to get to know you! You are a... = I was a researcher in the past, but moved to another career

As someone who left academia your opinion is also very important to us! **Please answer the rest of this survey thinking back at your time as a researcher.**

Example: Read '***Are you*** *affiliated with a Flemish University or scientific institute?*' as '***Were you*** *affiliated with a Flemish University or scientific institute?*'

Are you affiliated with a Flemish University or scientific institute?

- o Yes
- o No

Display This Question:
If Are you affiliated with a Flemish University or scientific institute? = Yes

Your main affiliation is at...

- o Hasselt University
- o VU Brussels
- o University of Antwerp
- o Ghent University
- o KU Leuven
- o IMEC
- o Institute of Tropical Medicine Antwerp
- o Other ____

Display This Question:
If Are you affiliated with a Flemish University or scientific institute? = No

Your main affiliation is at:

- o Institution ____
- o Country ____

You are in the Faculty of...

- o Medicine / Medicine and Health Sciences / Medicine and Life Sciences / Medicine and Pharmacy *(or equivalent)*
- o Other ____

And you have published ...

▼ ...fewer than 10 peer-reviewed papers ... ...over 210 peer-reviewed papers

Your gender is...

- Male
- Female
- Other
- Prefer not to answer

Are you currently working in your country of origin?

- No
- Yes

Display This Question:
If Are you currently working in your country of origin? = No

What is your country of origin?

▼ Prefer not to say / Afghanistan ... Zimbabwe

Display This Question:
If Are you currently working in your country of origin? = No

Have you worked as a researcher/research student in your country of origin?

- Yes
- No

Have you ever been involved in **evaluating researchers for promotion, tenure, or career advancement**?

- Yes
- No
- Not sure (explain) ____

End of Block: Demographics

Start of Block: Time management

Allright! Now we would like to know how you spend your time as a researcher.

Do you work full time as a researcher/PhD student?

- Yes
- No

Display This Question:
If Allright! Now we would like to know how you spend your time as a researcher. Do you work full tim... = No

Where would you situate yourself?

▼ less than 25% research employment ... over 75% but less than 100% research employment

On average, **HOW MANY HOURS** per week **do you work?** (overtime included)

I really work...

0 20 40 60 80

And during your work time, what **PERCENTAGE (%) of your time** do you spend on the following three pillars **in reality**, and **how would you like it to be**?

In reality...

PERCENTAGE (%) of your time

0 20 40 60 80 100

TEACHING
RESEARCH
OTHER

In my ideal world...

PERCENTAGE (%) of your time

0 20 40 60 80 100

TEACHING
RESEARCH
OTHER

And now a little more into the details:

What PERCENTAGE (%) of your time would you say you spend on average on each of the following activities **in reality**, and **how would you like it to be**?

In reality...

PERCENTAGE (%) of your time

0 20 40 60 80 100

DIRECT STUDENT SUPERVISION

HANDS-ON RESEARCH WORK (e.g., lab work, data analysis)

STAYING UP TO DATE (e.g., reading, listening, building skills, etc.)

WRITING PAPERS

REVIEWING

GRANT WRITING

ANYTHING ELSE (e.g., administration, meetings, etc.)

In your ideal world...

PERCENTAGE (%) of your time

	0	20	40	60	80	100
**DIRECT STUDENT SUPERVISION**						
**HANDS-ON RESEARCH WORK (e.g., lab work, data analysis)**						
**STAYING UP TO DATE (e.g., reading, listening, building skills, etc.)**						
**WRITING PAPERS**						
**REVIEWING**						
**GRANT WRITING**						
**ANYTHING ELSE (e.g., administration, meetings, etc.)**						

End of Block: Time management

Start of Block: Statements

Great! Now that we know each other, let's get to business!

In the following questions, we wish to know the impact of typical research activities
(A) on **advancing your career**
(B) on **advancing science**, and
(C) on **your personal satisfaction**

There will be **18 research activities** to rate. We numbered then from 18 to 1 so you know how many you have left!

18. Publishing papers is...

	essential	important	irrelevant	unfavorable	detrimental
**...in advancing my career**	○	○	○	○	○
**...in advancing science**	○	○	○	○	○
**...to my personal satisfaction**	○	○	○	○	○

Feel free to leave a comment (optional) ____

17. Publishing in high impact journals is...

	essential	important	irrelevant	unfavorable	detrimental
**...in advancing my career**	○	○	○	○	○
**...in advancing science**	○	○	○	○	○
**...to my personal satisfaction**	○	○	○	○	○

Feel free to leave a comment (optional) ____

16. Publishing commentaries or editorials is…

	essential	important	irrelevant	unfavorable	detrimental
**…in advancing my career**	○	○	○	○	○
**…in advancing science**	○	○	○	○	○
**…to my personal satisfaction**	○	○	○	○	○

Feel free to leave a comment (optional) ____

15. Publishing more papers than others is…

	essential	important	irrelevant	unfavorable	detrimental
**…in advancing my career**	○	○	○	○	○
**…in advancing science**	○	○	○	○	○
**…to my personal satisfaction**	○	○	○	○	○

Feel free to leave a comment (optional) ____

14. Publishing open access is…

	essential	important	irrelevant	unfavorable	detrimental
**…in advancing my career**	○	○	○	○	○
**…in advancing science**	○	○	○	○	○
**…to my personal satisfaction**	○	○	○	○	○

Feel free to leave a comment (optional) ____

13. Peer reviewing is…

	essential	important	irrelevant	unfavorable	detrimental
**…in advancing my career**	○	○	○	○	○
**…in advancing science**	○	○	○	○	○
**…to my personal satisfaction**	○	○	○	○	○

Feel free to leave a comment (optional) ____

12. Replicating past research is...

	essential	important	irrelevant	unfavorable	detrimental
**...in advancing my career**	○	○	○	○	○
**...in advancing science**	○	○	○	○	○
**...to my personal satisfaction**	○	○	○	○	○

Feel free to leave a comment (optional) ____

11. Publishing findings that did not work (i.e., negative findings) is...

	essential	important	irrelevant	unfavorable	detrimental
**...in advancing my career**	○	○	○	○	○
**...in advancing science**	○	○	○	○	○
**...to my personal satisfaction**	○	○	○	○	○

Feel free to leave a comment (optional) ____

10. Sharing your full data and detailed methods is...

	essential	important	irrelevant	unfavorable	detrimental
**...in advancing my career**	○	○	○	○	○
**...in advancing science**	○	○	○	○	○
**...to my personal satisfaction**	○	○	○	○	○

Feel free to leave a comment (optional) ____

9. Reviewing raw data from students and collaborators is...

	essential	important	irrelevant	unfavorable	detrimental
**...in advancing my career**	○	○	○	○	○
**...in advancing science**	○	○	○	○	○
**...to my personal satisfaction**	○	○	○	○	○

Feel free to leave a comment (optional) ____

8. Conducting innovative research with a high risk of failure is...

	essential	important	irrelevant	unfavorable	detrimental
**...in advancing my career**	○	○	○	○	○
**...in advancing science**	○	○	○	○	○
**...to my personal satisfaction**	○	○	○	○	○

Feel free to leave a comment (optional) ____

7. Connecting with renowned researchers is...

	essential	important	irrelevant	unfavorable	detrimental
**...in advancing my career**	○	○	○	○	○
**...in advancing science**	○	○	○	○	○
**...to my personal satisfaction**	○	○	○	○	○

Feel free to leave a comment (optional) ____

6. Collaborating across borders, disciplines, and sectors is...

	essential	important	irrelevant	unfavorable	detrimental
**...in advancing my career**	○	○	○	○	○
**...in advancing science**	○	○	○	○	○
**...to my personal satisfaction**	○	○	○	○	○

Feel free to leave a comment (optional) ____

5. Getting cited in scientific literature is...

	essential	important	irrelevant	unfavorable	detrimental
**...in advancing my career**	○	○	○	○	○
**...in advancing science**	○	○	○	○	○
**...to my personal satisfaction**	○	○	○	○	○

Feel free to leave a comment (optional) ____

4. Having your papers read and downloaded is...

	essential	important	irrelevant	unfavorable	detrimental
...in advancing my career	○	○	○	○	○
...in advancing science	○	○	○	○	○
...to my personal satisfaction	○	○	○	○	○

Feel free to leave a comment (optional) ____

3. Having public outreach (e.g., social media, news, etc.) is...

	essential	important	irrelevant	unfavorable	detrimental
...in advancing my career	○	○	○	○	○
...in advancing science	○	○	○	○	○
...to my personal satisfaction	○	○	○	○	○

Feel free to leave a comment (optional) ____

2. Having your results used or implemented in practice is...

	essential	important	irrelevant	unfavorable	detrimental
...in advancing my career	○	○	○	○	○
...in advancing science	○	○	○	○	○
...to my personal satisfaction	○	○	○	○	○

Feel free to leave a comment (optional) ____

1. Having luck is...

	essential	important	irrelevant	unfavorable	detrimental
...in advancing my career	○	○	○	○	○
...in advancing science	○	○	○	○	○
...to my personal satisfaction	○	○	○	○	○

Feel free to leave a comment (optional) ____

End of Block: Statements

Start of Block: End

Do you have any other comments or thoughts you would like to share with us? (optional)

____
____
___

End of Block: End
