# Supplementary material for: Advancing science or advancing careers? Researchers’ opinions on success indicators
Source: PLoS One. 2021 Feb 11;16(2):e0243664. doi: 10.1371/journal.pone.0243664 (PMC7878066; doi:10.1371/journal.pone.0243664)
Supplement: S1 Table — (PDF) [file pone.0243664.s004.pdf]

S1 TABLE

ANALYSIS RESULTS FOR RATINGS OF THE DIMENSIONAL IMPORTANCE OF THE 18 SUCCESS INDICATORS

Statement	Mean rating for each dimension	Result*	Bonferroni post hoc pairwise comparisons		
			Career vs Science	Career vs Satisfaction	Science vs Satisfaction
Publishing papers is...	Career: 4.52<br>Science: 4.37<br>Satisfaction: 3.90	(SA) F(2, 250) = 38.188<br>p < 0.001	95% CI(0.001, 0.301)<br>p = 0.048<br>md = 0.151	**95% CI(0.468, 0.786)<br>p < 0.001<br>md = 0.627**	**95% CI(0.341, 0.611)<br>p < 0.001<br>md = 0.476**
Publishing in high impact journals is...	Career: 4.31<br>Science: 3.73<br>Satisfaction: 3.67	(SA) F(2, 250) = 36.701<br>p < 0.001	**95% CI(0.418, 0.741)<br>p < 0.001<br>md = 0.579**	**95% CI(0.477, 0.809)<br>p < 0.001<br>md = 0.643**	95% CI(−0.100, 0.227)<br>p = 0.444<br>md = 0.063
Publishing commentaries or editorials is...	Career: 3.48<br>Science: 3.70<br>Satisfaction: 3.34	(SA) F(2, 250) = 14.538<br>p < 0.001	**95% CI(−0.360, −0.085)<br>p = 0.002<br>md = −0.222**	95% CI(0.000, 0.269)<br>p = 0.049<br>md = 0.135	**95% CI(0.232, 0.482)<br>p < 0.001<br>md = 0.357**
Publishing more papers than others is...	Career: 3.83<br>Science: 2.89<br>Satisfaction: 3.01	(GG) F(1.779, 222.388)<br>= 70.233<br>p < 0.001	**95% CI(0.761, 1.127)<br>p < 0.001<br>md = 0.944**	**95% CI(0.636, 1.015)<br>p < 0.001<br>md = 0.825**	95% CI(−0.258, 0.020)<br>p = 0.092<br>md = −0.119
Publishing open access is...	Career: 3.48<br>Science: 4.35<br>Satisfaction: 3.67	(SA) F(2, 250) = 62.624<br>p < 0.001	**95% CI(−1.034, −0.696)<br>p < 0.001<br>md = −0.865**	**95% CI(−0.357, −0.024)<br>p = 0.025<br>md = −0.190**	**95% CI(0.529, 0.821)<br>p < 0.001<br>md = 0.675**
Peer reviewing is...	Career: 3.39<br>Science: 4.43<br>Satisfaction: 3.47	(SA) F(2, 250) = 81.399<br>p < 0.001	**95% CI(−1.221, −0.858)<br>p < 0.001<br>md = −1.040**	95% CI(−0.260, 0.101)<br>p = 0.386<br>md = −0.079	**95% CI(0.784, 1.136)<br>p < 0.001<br>md = 0.960**
Replicating past research is...	Career: 2.83<br>Science: 3.98<br>Satisfaction: 3.09	(GG) F(1.847, 230.832)<br>= 81.530<br>p < 0.001	**95% CI(−1.360, −0.942)<br>p < 0.001<br>md = −1.151**	**95% CI(−0.442, −0.066)<br>p = 0.008<br>md = −0.254**	**95% CI(0.735, 1.059)<br>p < 0.001<br>md = 0.897**
Publishing findings that did not work (i.e., negative findings) is...	Career: 2.84<br>Science: 4.48<br>Satisfaction: 3.61	(GG) F(1.820, 227.487)<br>= 187.113<br>p < 0.001	**95% CI(−1.823, −1.462)<br>p < 0.001<br>md = −1.643**	**95% CI(−0.951, −0.588)<br>p < 0.001<br>md = −0.770**	**95% CI(0.734, 1.012)<br>p < 0.001<br>md = 0.873**
Sharing your full data and detailed methods is...	Career: 3.29<br>Science: 4.40<br>Satisfaction: 3.67	(GG) F(1.906, 238.310)<br>= 106.656<br>p < 0.001	**95% CI(−1.278, −0.960)<br>p < 0.001<br>md = −1.119**	**95% CI(−0.554, −0.224)<br>p < 0.001<br>md = −0.389**	**95% CI(0.594, 0.867)<br>p < 0.001<br>md = 0.730**
Reviewing raw data from students and collaborators is...	Career: 3.37<br>Science: 4.20<br>Satisfaction: 3.64	(SA) F(2, 250) = 50.707<br>p < 0.001	**95% CI(−0.992, −0.658)<br>p < 0.001<br>md = −0.825**	**95% CI(−0.446, −0.094)<br>p = 0.003<br>md = −0.270**	**95% CI(0.403, 0.708)<br>p < 0.001<br>md = 0.556**
Conducting innovative research with a high risks of failure is...	Career: 3.29<br>Science: 4.47<br>Satisfaction: 3.91	(GG) F(1.614, 201.766)<br>= 66.452<br>p < 0.001	**95% CI(−1.406, −0.943)<br>p < 0.001<br>md = −1.175**	**95% CI(−0.836, −0.402)<br>p < 0.001<br>md = −0.619**	**95% CI(0.410, 0.701)<br>p < 0.001<br>md = 0.556**
Connecting with renowned researchers is...	Career: 4.35<br>Science: 3.91<br>Satisfaction: 3.98	(SA) F(2, 250) = 24.566<br>p < 0.001	**95% CI(0.295, 0.578)<br>p < 0.001<br>md = 0.437**	**95% CI(0.238, 0.508)<br>p < 0.001<br>md = 0.373**	95% CI(−0.185, 0.058)<br>p = 0.304<br>md = −0.063
Collaborating across borders, disciplines, and sectors is...	Career: 4.25<br>Science: 4.64<br>Satisfaction: 4.36	(GG) F(1.558, 194.781)<br>= 14.565<br>p < 0.001	**95% CI(−0.550, −0.228)<br>p < 0.001<br>md = −0.389**	95% CI(−0.274, 0.068)<br>p = 0.235<br>md = −0.103	**95% CI(0.184, 0.388)<br>p < 0.001<br>md = 0.286**
Getting cited in scientific literature is...	Career: 4.46<br>Science: 3.66<br>Satisfaction: 3.98	(GG) F(1.890, 236.280)<br>= 55.630<br>p < 0.001	**95% CI(0.633, 0.970)<br>p < 0.001<br>md = 0.802**	**95% CI(0.345, 0.623)<br>p < 0.001<br>md = 0.484**	**95% CI(−0.463, −0.172)<br>p < 0.001<br>md = −0.317**

Having your papers read and downloaded is…	Career: 3.90<br>Science: 3.90<br>Satisfaction: 4.10	(SA) $F(2, 250) = 4.873$<br>$p = 0.008$	95% CI(−0.158, 0.158)<br>$p = 1.000$<br>md = 0.000	**95% CI(−0.365, −0.048)**<br>**$p = 0.011$**<br>**md = −0.206**	**95% CI(−0.343, −0.070)**<br>**$p = 0.003$**<br>**md = −0.206**
Having public outreach (e.g., social media, news, etc.) is…	Career: 3.84<br>Science: 3.77<br>Satisfaction: 3.72	(SA) $F(2, 250) = 1.251$<br>$p = 0.288$	———	———	———
Having your results used or implemented in practice is…	Career: 4.02<br>Science: 4.26<br>Satisfaction: 4.37	(SA) $F(2, 250) = 12.875$<br>$p < 0.001$	**95% CI(−0.380, −0.096)**<br>**$p = 0.001$**<br>**md = −0.238**	**95% CI(−0.479, −0.203)**<br>**$p < 0.001$**<br>**md = −0.341**	95% CI(−0.233, 0.027)<br>$p = 0.118$<br>md = −0.103
Having luck is…	Career: 4.27<br>Science: 4.02<br>Satisfaction: 3.89	(SA) $F(2, 250) = 14.229$<br>$p < 0.001$	**95% CI(0.115, 0.377)**<br>**$p < 0.001$**<br>**md = 0.246**	**95% CI(0.240, 0.522)**<br>**$p < 0.001$**<br>**md = 0.381**	95% CI(−0.022, 0.292)<br>$p = 0.091$<br>md = 0.135

* We report Greenhouse-Geisser (GG) tests when the results of Mauchly's test of sphericity could not confirm the sphericity of the data. Otherwise, Sphericity Assumed (SA) tests are reported. Abbreviations: confidence intervals (CI), mean difference (md)
