# Supplementary material for: Advancing science or advancing careers? Researchers’ opinions on success indicators
Source: PLoS One. 2021 Feb 11;16(2):e0243664. doi: 10.1371/journal.pone.0243664 (PMC7878066; doi:10.1371/journal.pone.0243664)
Supplement: Attachment — Submitted filename: PONE-D-20-19974_ResponseToReviewer.pdf [file pone.0243664.s005.pdf]

INTRODUCTORY NOTE FROM THE AUTHORS:

Dear Dr. Nunes Amaral, dear Prof. Waltman,

Thank you very much for your careful review of our manuscript. I really appreciate the excellent comments of Prof. Waltman, and I thoroughly adapted the manuscript to fit all points raised.

Being a PhD student with little experience in reporting confidence intervals however, I wanted to make sure that I was doing the changes correctly. For this reason, I detail what I did below, and would be grateful if I could receive a bit of feedback on my adaptations to feel comfortable that our findings are reported appropriately.

In short, I re-ran the analyses to capture the confidence intervals and added those throughout to a new results table (Table 4) and to the Supplementary table S4. For the time analyses I grabbed the lower bound and the upper bound directly from R and rounded them to second digit. For the dimensions analyses, I took the confidence intervals that are reported alongside the 'Pairwise Comparisons' (i.e., the post hoc tests) in SPSS, and reported them in the Supplementary file S4 keeping all three digits reported (I could have reduced to 2 digits but I was unsure what was best). Throughout the manuscript, I removed statistical data and referred readers to Table 4 or Supplementary Table S4. I rounded p values to <0.05, <0.01, and <0.001 to avoid the false sense of precision, and I added the corresponding significance (*, **, ***, respectively) in the new Fig. 1. The reason I took out all values from the text was because I was concurrently adapting the manuscript to reviewers of my thesis who suggested the change. I am happy to adapt to whatever you find best.

Please let me know if there is anything I could have done better. I am happy to edit the manuscript further (that is, after my thesis defence of the 14th!).

I sincerely thank both of you for your time and efforts. I am learning in the process of this project, and I feel very fortunate to benefit from your expertise. I also added a note to thank Prof. Waltman in the acknowledgements, if he prefers not to be mentioned I can take it out.

I look forward to hearing back from you.

Kind regards,
Noémie Aubert Bonn (Corresponding author)

PONE-D-20-19974

Advancing science or advancing careers? Researchers' opinions on success indicators

PLOS ONE

Dear Dr. Aubert Bonn,

Thank you for submitting your manuscript to PLOS ONE. After careful consideration, we feel that it has merit but does not fully meet PLOS ONE's publication criteria as it currently stands. Therefore, we invite you to submit a revised version of the manuscript that addresses the points raised during the review process.

Please change the discussion of the results and implications according to the notes of the reviewer (see paragraph starting with "In the final section, the authors draw a few quite strong ..."

RESPONSE FROM THE AUTHORS: Thank you, I have changed the text according to the reviewer's excellent comments. My specific changes are detailed below and visible in the manuscript that contains track changes.

Also, present all numerical estimates for parameters with just the SIGNIFICANT digits. Which digits are significant should be apparent from CI for estimates.

RESPONSE FROM THE AUTHORS: I have adapted the reporting of statistical values, but am unsure if my reporting of significant digits (as mentioned above). I hope that I did it correctly, and am very happy to edit as needed.

Please submit your revised manuscript by Oct 05 2020 11:59PM. If you will need more time than this to complete your revisions, please reply to this message or contact the journal office at plosone@plos.org. When you're ready to submit your revision, log on to https://www.editorialmanager.com/pone/ and select the 'Submissions Needing Revision' folder to locate your manuscript file.

Please include the following items when submitting your revised manuscript:

- A rebuttal letter that responds to each point raised by the academic editor and reviewer(s). You should upload this letter as a separate file labeled 'Response to Reviewers'.
- A marked-up copy of your manuscript that highlights changes made to the original version. You should upload this as a separate file labeled 'Revised Manuscript with Track Changes'.
- An unmarked version of your revised paper without tracked changes. You should upload this as a separate file labeled 'Manuscript'.

If you would like to make changes to your financial disclosure, please include your updated statement in your cover letter. Guidelines for resubmitting your figure files are available below the reviewer comments at the end of this letter.

If applicable, we recommend that you deposit your laboratory protocols in protocols.io to enhance the reproducibility of your results. Protocols.io assigns your protocol its own identifier (DOI) so that it can be cited independently in the future. For instructions see: http://journals.plos.org/plosone/s/submission-guidelines#loc-laboratory-protocols

We look forward to receiving your revised manuscript.

Kind regards,

Luís A. Nunes Amaral, Ph.D.

Academic Editor

PLOS ONE

Journal Requirements:

When submitting your revision, we need you to address these additional requirements.

1. Please ensure that your manuscript meets PLOS ONE's style requirements, including those for file naming. The PLOS ONE style templates can be found at

https://journals.plos.org/plosone/s/file?id=wjVg/PLOSOne_formatting_sample_main_body.pdf and

https://journals.plos.org/plosone/s/file?id=ba62/PLOSOne_formatting_sample_title_authors_affiliations.pdf

2. Please include additional information regarding the survey or questionnaire used in the study and ensure that you have provided sufficient details that others could replicate the analyses.

For instance, if you developed a questionnaire as part of this study and it is not under a copyright license more restrictive than CC-BY, please include a copy, in both the original language and English, as Supporting Information.

3. Please include captions for your Supporting Information files at the end of your manuscript, and update any in-text citations to match accordingly. Please see our Supporting Information guidelines for more information: http://journals.plos.org/plosone/s/supporting-information

4. Your ethics statement must appear in the Methods section of your manuscript. If your ethics statement is written in any section besides the Methods, please move it to the Methods section and delete it from any other section. Please also ensure that your ethics statement is included in your manuscript, as the ethics section of your online submission will not be published alongside your manuscript.

[Note: HTML markup is below. Please do not edit.]

Reviewers' comments:

Reviewer's Responses to Questions

Comments to the Author

1. Is the manuscript technically sound, and do the data support the conclusions?

The manuscript must describe a technically sound piece of scientific research with data that supports the conclusions. Experiments must have been conducted rigorously, with appropriate controls, replication, and sample sizes. The conclusions must be drawn appropriately based on the data presented.

Reviewer #1: Yes

---

2. Has the statistical analysis been performed appropriately and rigorously?

Reviewer #1: Yes

---

3. Have the authors made all data underlying the findings in their manuscript fully available?

The PLOS Data policy requires authors to make all data underlying the findings described in their manuscript fully available without restriction, with rare exception (please refer to the Data Availability Statement in the

manuscript PDF file). The data should be provided as part of the manuscript or its supporting information, or deposited to a public repository. For example, in addition to summary statistics, the data points behind means, medians and variance measures should be available. If there are restrictions on publicly sharing data—e.g. participant privacy or use of data from a third party—those must be specified.

Reviewer #1: Yes

---

4. Is the manuscript presented in an intelligible fashion and written in standard English?

PLOS ONE does not copyedit accepted manuscripts, so the language in submitted articles must be clear, correct, and unambiguous. Any typographical or grammatical errors should be corrected at revision, so please note any specific errors here.

Reviewer #1: Yes

---

5. Review Comments to the Author

Please use the space provided to explain your answers to the questions above. You may also include additional comments for the author, including concerns about dual publication, research ethics, or publication ethics. (Please upload your review as an attachment if it exceeds 20,000 characters)

Reviewer #1: I recommend to accept this paper for publication after the authors have made some small improvements.

On p. 8, the authors state that “125 participants completed the survey”. However, according to Table 2, there are 126 participants.

RESPONSE FROM THE AUTHORS: Thank you for noticing this mistake, it should indeed read 126.

“On average, respondents who declared working full time worked 46.91 hours per week (median 46) but the distribution was very wide (Table 3)” (p. 8): Table 3 doesn’t show that the distribution is very wide. The table shows a maximum value, but this is insufficient to conclude that a distribution is very wide.

RESPONSE FROM THE AUTHORS: Correct. I added the minimum values to Table 3 so the range can be observed.

“we found that participants wished they could dedicate more time to ‘Teaching’ (paired t(92) = 3.3539, p = 0.001159) and especially to ‘Research’ (paired t(92) = 4.2818, p = 4.545e-05)” (p. 10): I don’t understand the result for teaching. Looking at the results for teaching in Figure 1, it seems that ‘reality’ and ‘ideal’ more or less coincide, and if there is a difference, it seems that ‘ideal’ is below ‘reality’, not above.

RESPONSE FROM THE AUTHORS: I am very grateful that you spotted this and pointed it out to me. It took me some time to understand what was happening, but I then realized that the format of Fig. 1 as I submitted it was incorrect (it only considered unique values and therefore reported incorrect medians and standard deviations). I have now rectified this problem and have thoroughly updated Fig. 1. I also added a green line to represent the mean for each category to allow better comparison. I double-checked the accuracy of the reported means and medians. I hope the new figure is clearer and more informative, and I sincerely thank you again for spotting this issue before publication.

“Almost 80% of full-time researchers who responded to our survey worked more than 40 hours per week” (p. 15): This statement is not entirely accurate. It would be more accurate to write: “Almost 80% of full-time researchers who responded to our survey report to work more than 40 hours per week”. I wonder whether people are able to accurately estimate the number of hours they work per week. Personally I find it quite difficult to estimate this. It could be that people systematically overestimate or underestimate the number of hours they work. Perhaps the

authors could check whether there is any literature in which the accuracy of these types of survey responses is investigated.

<u>RESPONSE FROM THE AUTHORS</u>: Absolutely. I adapted the text to capture this distinction, and also changed the title of Fig. 1 to "Self-reported percentage of time". I also looked for some literature on the topic and added the following paragraph to the Limitations section:

"Second, asking participants to estimate their working hours and time distribution relies on precision of recall and accuracy of self-report; two aspects we had no means to verify in the current work. Assessments of the reliability of self-reported working hours are largely absent from the literature. We only found one published paper to support the correlation between recorded and self-reported working hours, but it concerned Japanese workers highly aware of their working schedules (14). We cannot assume that similar findings would be observed in academic researchers whose working hours vary greatly and whose task concentration changes between academic year periods. Past works and popular surveys investigating the time allocation of scientists found different distribution of work allocation than those we found in our survey, generally reporting a higher proportion of time spent teaching (15, 16). This difference, which is probably due to the high representation of PhD students among our participants, suggests that our findings might not be representative of other settings and populations and should be interpreted with caution. Despite this limitation, our findings coincide with other works in stating that researchers report working overtime (15, 17-21), that they are subject to heavy administrative burden (21, 22), and that they wish they could dedicate more time to research (23).

14. Imai T, Kuwahara K, Miyamoto T, Okazaki H, Nishihara A, Kabe I, et al. Validity and reproducibility of self-reported working hours among Japanese male employees. J Occup Health. 2016;58(4):340-6.
15. Ziker J. The Blue Review. 2014 31 March. Available from: https://www.boisestate.edu/bluereview/faculty-time-allocation/.
16. Matthews D. If you love research, academia may not be for you. Times Higher Education. 2018 8 November. Available from: https://www.timeshighereducation.com/blog/if-you-love-research-academia-may-not-be-you.
17. Barnett A, Mewburn I, Schroter S. Working 9 to 5, not the way to make an academic living: observational analysis of manuscript and peer review submissions over time. BMJ. 2019;367.
18. Powell K. Young, talented and fed-up: scientists tell their stories. Nature. 2016;538:446-9.
19. Bothwell E. Work-life balance survey 2018: long hours take their toll on academics. Times Higher Education. 2018 8 February. Available from: https://www.timeshighereducation.com/features/work-life-balance-survey-2018-long-hours-take-their-toll-academics.
20. Mckenna L. How Hard Do Professors Actually Work? The Atlantic. 2018 7 February. Available from: https://www.theatlantic.com/education/archive/2018/02/how-hard-do-professors-actually-work/552698/.
21. Koens L, R., Jonge HaJd. What motivates researchers? Research excellence is still a priority. The Hague: Rathenau Instituut; 2018. Available from: https://www.rathenau.nl/sites/default/files/2018-07/What%20motivates%20researchers.pdf.
22. Schneider SL, Ness KK, Shaver K, Brutkiewicz R. Federal Demonstration Partnership 2012 Faculty Workload Survey - Research Report. 2014. Available from: https://osp.od.nih.gov/wp-content/uploads/SMRB_May_2014_2012_Faculty_Workload_Survey_Research_Report.pdf.
23. Mergaert L, Raeymaekers P. Researchers at Belgian Universities: What drives them? Which obstacles do they encounter? : Kind Baudouin Foundation; 2017. Report No.: ISBN: D/2893/2017/16. Available from: https://www.kbs-frb.be/en/Virtual-Library/2017/20171113PP."

In the final section, the authors draw a few quite strong conclusions based on their survey (e.g., "a thorough restructuration of the resources and infrastructures needs to take place"). While these conclusions seem reasonable to me, I think the authors need to acknowledge that the survey provides only limited evidence to support these conclusions. There are various reasons why the evidence provided by the survey is limited. First, the number of survey participants is quite limited. Second, there probably is a participation bias. Third, most respondents are from Flanders, so the survey can be used to draw conclusions only about Flanders. My recommendation to the authors is to emphasize how the survey results align with the broader literature. In this way, the authors can make clear that their conclusions are supported not only by their survey results, but also by the broader literature.

<u>RESPONSE FROM THE AUTHORS</u>: Thank you for noticing this point. I have added additional examples from the literature within the discussion, and I have toned down the conclusion by noting the following:

"It is important to consider that our survey captured the perspectives of a limited sample of predominantly Flemish researchers and may thus be of limited generalisability. Nonetheless, our findings align with a growing body of international works, declarations, and reports on the topic (see for example 2, 3, 35, 36, 43, 44).

Together with this growing body of literature, our findings support that research assessments need to be addressed so that researchers' careers consider activities that pursue the genuine advancement of science."

I am happy to adapt it further if deemed necessary.

The authors sometimes present numerical results with lots of decimals (e.g., bottom paragraph on p. 10). There is no need to report so many decimals. These decimals are not informative. My recommendation is to reduce the number of decimals that are reported.

RESPONSE FROM THE AUTHORS: As mentioned in the introductory text, I have moved all statistical results from this section to Table 4. I only keot two decimals and have simplified the p values throughout to $p < 0.05$, $p < 0.01$, and $p < 0.001$. Note that I left all three decimals in Supplementary Table S4. I would be happy to implement any changes that seems fit.

Following initiatives to improve statistical reporting (e.g., https://en.wikipedia.org/wiki/Estimation_statistics), I believe it would be preferable to focus more strongly on measures of effect size (e.g., the mean difference in the answers given to different survey questions), complemented with confidence intervals, instead of using null hypothesis significance testing (NHST). NHST has the disadvantage of promoting dichotomous ways of thinking. The magnitude of an effect often doesn't get proper attention in NHST. Since there is no agreement on the pros and cons of different statistical methods, I consider the use of NHST to be acceptable, but I hope the authors will consider using more appropriate statistical methods in the future.

RESPONSE FROM THE AUTHORS: As mentioned in the 'Introductory note from the authors' above, we are learning in this process and are grateful to learn the best methods. I re-ran the analyses to capture the confidence intervals and added those throughout Table 4 and the Supplementary table S4. As noted above, one of the jury of my thesis suggested that it may be better to take out the statistical analyses entirely from this manuscript, and this explains why I moved the results to Table 4. I would appreciate your views on this matter.

---

6. PLOS authors have the option to publish the peer review history of their article (what does this mean?). If published, this will include your full peer review and any attached files.

If you choose "no", your identity will remain anonymous but your review may still be made public.

Do you want your identity to be public for this peer review? For information about this choice, including consent withdrawal, please see our Privacy Policy.

Reviewer #1: **Yes:** Ludo Waltman

[NOTE: If reviewer comments were submitted as an attachment file, they will be attached to this email and accessible via the submission site. Please log into your account, locate the manuscript record, and check for the action link "View Attachments". If this link does not appear, there are no attachment files.]

While revising your submission, please upload your figure files to the Preflight Analysis and Conversion Engine (PACE) digital diagnostic tool, https://pacev2.apexcovantage.com/. PACE helps ensure that figures meet PLOS requirements. To use PACE, you must first register as a user. Registration is free. Then, login and navigate to the UPLOAD tab, where you will find detailed instructions on how to use the tool. If you encounter any issues or have any questions when using PACE, please email PLOS at figures@plos.org. Please note that Supporting Information files do not need this step.
