# Supplementary material for: Advancing science or advancing careers? Researchers’ opinions on success indicators
Source: PLoS One. 2021 Feb 11;16(2):e0243664. doi: 10.1371/journal.pone.0243664 (PMC7878066; doi:10.1371/journal.pone.0243664)
Supplement: Attachment — Submitted filename: Response to editor.pdf [file pone.0243664.s006.pdf]

Response to editor and reviewer **15 November 2020**
Manuscript PONE-D-20-19974R1

Dear Luís A. Nunes Amaral, Dear Prof. Waltman,

I wish to thank you both for your help and patience, and for allowing me learn how to better report my findings. I have now adapted the manuscript and accompanying files to reflect the changes we discussed. My changes are as follows:

Changes to the manuscript:

- **I corrected Table 4 by performing all analyses without the log correction. Most results remained the same, but the difference between real and ideal times spent 'Writing grants' became significant as a result of the change (i.e., respondents wish they could spend *less* time writing grants). I believe that this is due to the skewed response curve in the time spent writing grant, possibly because of the different seniority profiles included in the study. For instance, PhD students are likely to spend very little time writing grants, while early career researchers sometime spend the majority of their time doing it. While the log transformation would control for this skewed distribution of answers, the new findings do not and the means are now interpreted as significantly different. To reflect this finding, I added a small section in the Limitation section stating**
    - ***"…we also found important to point out that the diversity of profiles included in our study may have impacted the distribution of answers. For example, the amount of time that a PhD student spends writing grants is likely to differ substantially from the amount of time an early career or tenured researcher spends on the same task. In our findings, we aggregated the groups to obtain a general portrait of the time distribution of researchers, but we invite readers to use the data provided if they wish to assess specific differences between seniority profiles."***

- **I adapted the rest of the text to reflect the new finding about the time spent writing grants**

- **I also realised that I did not mention the statistical softwares used and added those to the Methods-Analysis section.**

- **Finally, I did a few changes to quotation marks (' vs ") since I noticed that I was not consistent in their use.**

Changes to figures and accompanying files:

- **I adapted Figure 1 to reflect new statistical significance (*, **, ***)**

- **I adapted Supplementary file S2 in which I report the Code used to reflect the new statistical code used. In doing so, I also added a small section to create the files to be used in the analyses from the supplementary data file so that the results are easily reusable.**

I look forward to hearing back from you.

Kind regards,
Noémie

PONE-D-20-19974R1
Advancing science or advancing careers? Researchers' opinions on success indicators
PLOS ONE

Dear Dr. Aubert Bonn,

Thank you for submitting your manuscript to PLOS ONE. After careful consideration, we feel that it has merit but does not fully meet PLOS ONE's publication criteria as it currently stands. Therefore, we invite you to submit a revised version of the manuscript that addresses the points raised during the review process.

As we discussed over email, please revise the reporting of the results in Table 4 according to the procedure we agreed upon.

Please submit your revised manuscript by Dec 27 2020 11:59PM. If you will need more time than this to complete your revisions, please reply to this message or contact the journal office at plosone@plos.org. When you're ready to submit your revision, log on to https://www.editorialmanager.com/pone/ and select the 'Submissions Needing Revision' folder to locate your manuscript file.

Please include the following items when submitting your revised manuscript:

- A rebuttal letter that responds to each point raised by the academic editor and reviewer(s). You should upload this letter as a separate file labeled 'Response to Reviewers'.
- A marked-up copy of your manuscript that highlights changes made to the original version. You should upload this as a separate file labeled 'Revised Manuscript with Track Changes'.
- An unmarked version of your revised paper without tracked changes. You should upload this as a separate file labeled 'Manuscript'.

If you would like to make changes to your financial disclosure, please include your updated statement in your cover letter. Guidelines for resubmitting your figure files are available below the reviewer comments at the end of this letter.

If applicable, we recommend that you deposit your laboratory protocols in protocols.io to enhance the reproducibility of your results. Protocols.io assigns your protocol its own identifier (DOI) so that it can be cited independently in the future. For instructions see: http://journals.plos.org/plosone/s/submission-guidelines#loc-laboratory-protocols

We look forward to receiving your revised manuscript.

Kind regards,

Luís A. Nunes Amaral, Ph.D.
Academic Editor
PLOS ONE

[Note: HTML markup is below. Please do not edit.]

Reviewers' comments:

Reviewer's Responses to Questions

Comments to the Author

1. If the authors have adequately addressed your comments raised in a previous round of review and you feel that this manuscript is now acceptable for publication, you may indicate that here to bypass the "Comments to the Author" section, enter your conflict of interest statement in the "Confidential to Editor" section, and submit your "Accept" recommendation.

Reviewer #1: (No Response)

---

2. Is the manuscript technically sound, and do the data support the conclusions?

The manuscript must describe a technically sound piece of scientific research with data that supports the conclusions. Experiments must have been conducted rigorously, with appropriate controls, replication, and sample sizes. The conclusions must be drawn appropriately based on the data presented.

Reviewer #1: Yes

---

3. Has the statistical analysis been performed appropriately and rigorously?

Reviewer #1: No

---

4. Have the authors made all data underlying the findings in their manuscript fully available?

The PLOS Data policy requires authors to make all data underlying the findings described in their manuscript fully available without restriction, with rare exception (please refer to the Data Availability Statement in the manuscript PDF file). The data should be provided as part of the manuscript or its supporting information, or deposited to a public repository. For example, in addition to summary statistics, the data points behind means, medians and variance measures should be available. If there are restrictions on publicly sharing data—e.g. participant privacy or use of data from a third party—those must be specified.

Reviewer #1: Yes

---

5. Is the manuscript presented in an intelligible fashion and written in standard English?

PLOS ONE does not copyedit accepted manuscripts, so the language in submitted articles must be clear, correct, and unambiguous. Any typographical or grammatical errors should be corrected at revision, so please note any specific errors here.

Reviewer #1: Yes

---

6. Review Comments to the Author

Please use the space provided to explain your answers to the questions above. You may also include additional comments for the author, including concerns about dual publication, research ethics, or publication ethics. (Please upload your review as an attachment if it exceeds 20,000 characters)

Reviewer #1: Thank you for the careful revision of your paper. I consider your work to be acceptable for publication. However, I find it hard to understand the confidence intervals reported in Table 4. I recommend to remove them from your paper.

---

7. PLOS authors have the option to publish the peer review history of their article (what does this mean?). If published, this will include your full peer review and any attached files.

If you choose “no”, your identity will remain anonymous but your review may still be made public.

Do you want your identity to be public for this peer review? For information about this choice, including consent withdrawal, please see our Privacy Policy.

Reviewer #1: **Yes:** Ludo Waltman

[NOTE: If reviewer comments were submitted as an attachment file, they will be attached to this email and accessible via the submission site. Please log into your account, locate the manuscript record, and check for the action link "View Attachments". If this link does not appear, there are no attachment files.]

While revising your submission, please upload your figure files to the Preflight Analysis and Conversion Engine (PACE) digital diagnostic tool, https://pacev2.apexcovantage.com/. PACE helps ensure that figures meet PLOS requirements. To use PACE, you must first register as a user. Registration is free. Then, login and navigate to the UPLOAD tab, where you will find detailed instructions on how to use the tool. If you encounter any issues or have any questions when using PACE, please email PLOS at figures@plos.org. Please note that Supporting Information files do not need this step.

In compliance with data protection regulations, you may request that we remove your personal registration details at any time. (Remove my information/details). Please contact the publication office if you have any questions.
